# Re-assembly of nineteenth-century smallpox vaccine genomes reveals the contemporaneous use of horsepox and horsepox-related viruses in the USA

**DOI:** 10.1186/s13059-020-02202-0

**Published:** 2020-12-04

**Authors:** Annika Brinkmann, Aline R. V. Souza, José Esparza, Andreas Nitsche, Clarissa R. Damaso

**Affiliations:** 1 Centre for Biological Threats and Special Pathogens 1 – Highly Pathogenic Viruses & German Consultant Laboratory for Poxviruses & WHO Collaborating Centre for Emerging Infections and Biological Threats, Robert Koch Institute, Berlin, Germany; 2 Instituto de Biofísica Carlos Chagas Filho, Universidade Federal do Rio de Janeiro, Av. Carlos Chagas Filho, 373 - CCS, Ilha do Fundão, Rio de Janeiro, RJ 21941-590 Brazil; 3 Institute of Human Virology, University of Maryland School of Medicine, Baltimore, MD USA

## Abstract

According to a recent article published in *Genome Biology*, Duggan and coworkers sequenced and partially assembled five genomes of smallpox vaccines from the nineteenth century. No information regarding the ends of genomes was presented, and they are important to understand the evolutionary relationship of the different smallpox vaccine genomes during the centuries. We re-assembled the genomes, which include the largest genomes in the vaccinia lineage and one true horsepox strain. Moreover, the assemblies reveal a diverse genetic structure in the genome ends. Our data emphasize the concurrent use of horsepox and horsepox-related viruses as the smallpox vaccine in the nineteenth century.

To the Editor

It is still a mystery which virus early vaccinators and vaccine manufacturers used as the smallpox vaccine in the nineteenth century, whether it was cowpox (CPXV), horsepox (HSPV), or vaccinia virus (VACV). Edward Jenner, who developed the first smallpox vaccine in 1796, supposedly used cowpox lymph but historical evidence accounts for the use of horsepox lymph on several occasions, including his first immunization experiments [1–3]. In fact, CPXV has never been detected molecularly in any smallpox vaccine. However, an HSPV-related virus has recently been described as the smallpox vaccine seed used by the Mulford Laboratories in the USA in 1902 [4].

The Mulford 1902 genome is > 99.7% similar to the central conserved region of the HSPV-MNR-76 genome. However, it differs in the variable flanking regions, mainly by the presence of two deletions of 10.7 kb and 5.5 kb in the left and right genome ends, respectively, which are a hallmark of all known VACV strains [4, 5]. Therefore, the analysis of the whole genome structure is essential to understand the genetic makeup of old smallpox vaccines [6].

In a recent *Genome Biology* article, Duggan and colleagues described the partial genomic sequences of five American smallpox vaccines from the mid to late nineteenth century [7]. Phylogenetic analyses revealed that the viruses are closely related to HSPV and to the Mulford 1902 strain. However, the only genome assembled de novo (VK1) has 184,677 bp and lacks nearly 20,000 bp of the left end. Because the right end is complete, we hypothesized that reads covering the left end should also be available.

Therefore, FastQ files were downloaded from Sequence Read Archive (PRJNA561155) and trimmed (Trimmomatic-v0.39, Phred-33 quality score) [8]. Full genomes were assembled by an iterative workflow: de novo assembly of adapter-removed reads by using Spades v3.13.1 (Phred offset-33, standard parameters) [9], mapping of the trimmed reads to the contigs to increase contig size, visual screening for accuracy, and correction of mis-assembled regions with Geneious Prime 2020.0.5. The final genomes were validated for accuracy by mapping with all reads and screened for inconsistency in the continuous assembly. Inverted terminal repeat (ITR) regions were identified with Geneious Prime Repeat Finder. Genomes were annotated by using Genome Annotation Transfer Utility (GATU) [10] and CLC Main Workbench v8.0, followed by visual screening [4, 6]. Orthopoxvirus sequences were aligned by using Mafft Server v7 [11] and used for phylogenetic inference by using Mega v6 [12].

All five re-assembled genomes are phylogenetically clustered within the HSPV subgroup of the VACV lineage (Fig. 1), confirming the findings of Duggan and colleagues [7]. However, our data provides important genetic information that was not revealed by the published assembly. We observed genomes of different sizes and number of ORFs and, interestingly, with distinct structures in the left and right ends. Table 1 summarizes our findings and Fig. 2 shows the genome structure of the left and right ends of the VK genomes. VK01 and VK12 have the largest genomes in the VACV lineage with 214,388 bp and 219,647 bp (Table 1), respectively, mainly due to the presence of unique insertions of 14.2 kb and 15.8 kb in the left end, probably resulting from a non-tandem duplication of an equivalent region in the right end of the genome and the insertion of cowpox gene orthologs (Fig. 2a, insert).

**Fig. 1:**
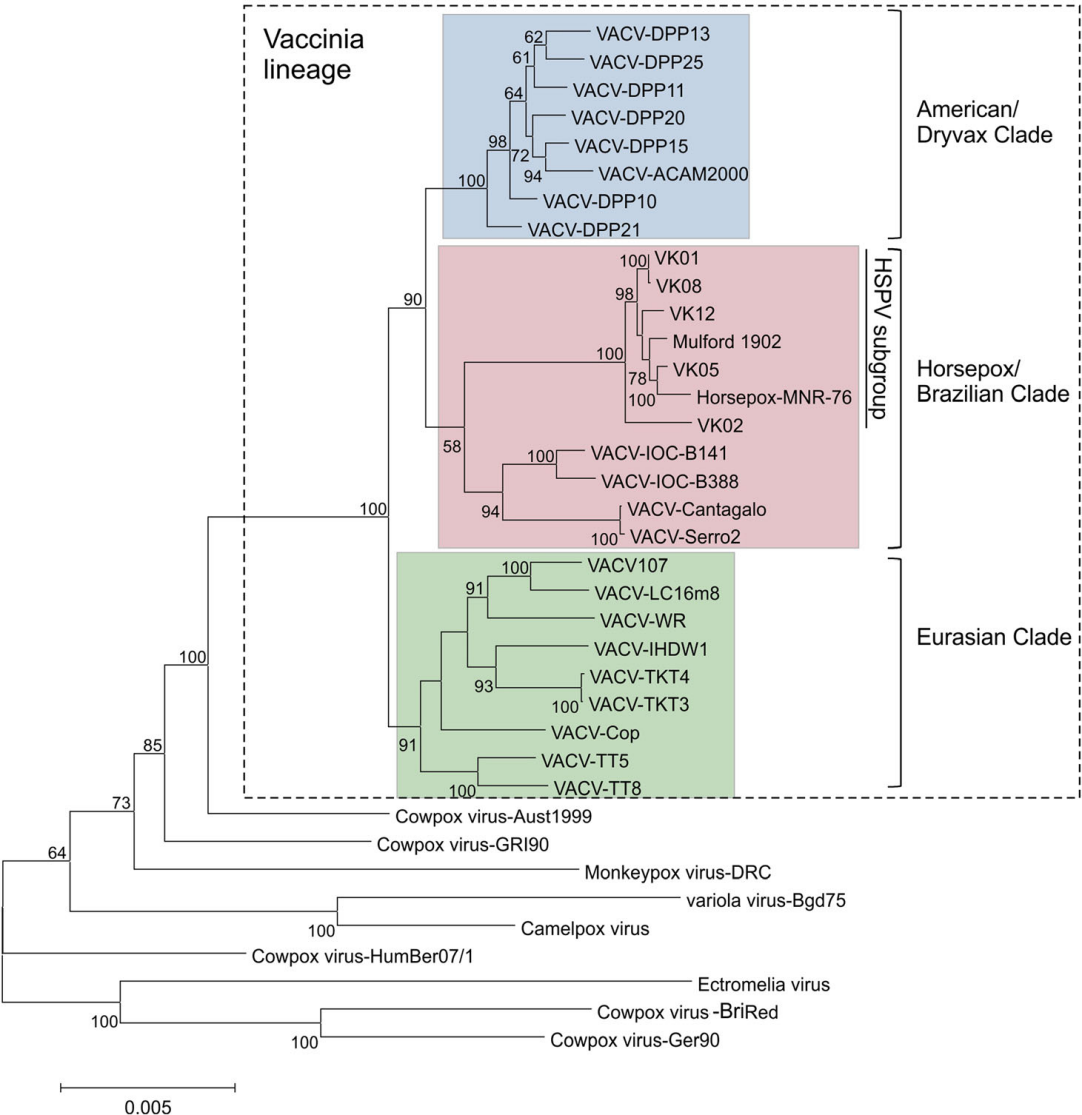
Phylogenetic inference of the old smallpox vaccines VK01, VK02, VK05, VK08, and VK12. The multi-alignment of 37 orthopoxvirus genomes, including the VK samples, was used as input for tree construction by using MEGA 6, opting for the maximum likelihood method based on the Tamura-Nei substitution model, Uniform rates model with 1000 bootstrap replicates. Numbers indicate the percentage of bootstrap support from 1000 replicates (> 50% is shown). The scale bar indicates the number of substitutions per site. The VACV clusters are indicated on the right. A similar tree topology was obtained by using the neighbor-joining method. GenBank accession numbers are indicated in the “Availability of data and materials” section

**Table 1:** Genomic features of the re-assembled genomes of VK01, VK02, VK05, VK08, and VK12 vaccines

Sample ^a^(year)		Genome size (bp)	ITR size (bp)	ORFs (no.)	Genome coverage (fold)	% identity HSPV MNR-76		^c^Presence of the deletions in the genome’s left and right ends characteristic of all VACV strains but absent in HSPV
						Whole genome	^b^Conserved core region	
Horsepox-related virus with VACV-like genome ends	VK01 (1866)	214,388	22,960	248	1762	0.903	0.997	Yes
	VK08 (1873)	204,481	9070	240	746	0.943	0.997	Yes
Horsepox-related virus with one genome end similar to VACV	VK02 (ND)	199,509	616	236	662	0.889	0.995	Deletion in the left end only
	VK12 (1859)	219,647	23,165	253	937	0.856	0.997	Deletion in the right end only
Horsepox virus	VK05 (ND)	212,688	6935	236	946	0.997	0.998	No
	^d^HSPV MNR-76	212,633	7527	236	ND	1	1	No

^a^Years according to Duggan et al. [7]. ND means not determined

^b^The conserved core region refers to approximately 99.0000 bp spanning from genes F9L to A24R

^c^The deletions correspond to 10.7 kb and 5.5 kb stretches of DNA present in HSPV-MR76, but absent in all VACV strains and in the Mulford 1902 [4, 5]

^d^Horsepox virus strain MNR-76 was included for the sake of comparison [5]. GenBank accession numbers are indicated in the “Availability of data and materials” section

**Fig. 2:**
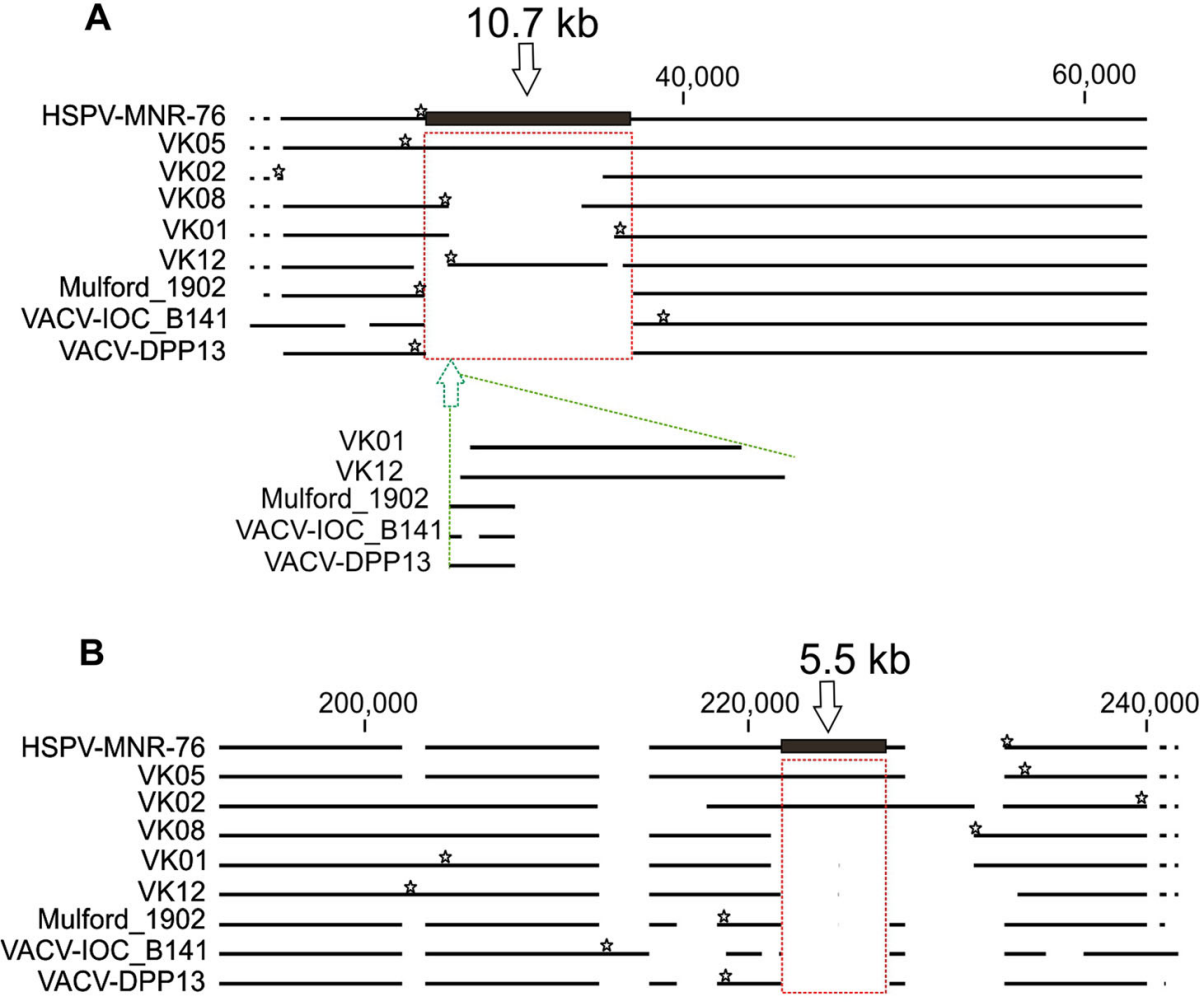
Diagram of the terminal regions of the genomes of the old smallpox vaccines VK01, VK02, VK05, VK08, and VK12. The genomes of the VK vaccines, horsepox MNR-76, Mulford 1902 vaccine, VACV-IOC_B141, and Dryvax clone DPP13 were aligned using the Geneious alignment plugin in Geneious Prime and visualized in CLC Main Workbench. A schematic figure was created based on the alignment showing the left (**a**) and the right (**b**) termini of the genomes. Minor deletions and gaps were omitted. For sake of clarity, insertions in the genomes of VK01, VK12, Mulford 1902, VACV-IOC_B141, and VACV-DPP13 that split the 10.7- kb sequence of horsepox genome in two parts were removed and are shown as in insert on the bottom (green dotted line) of **a**. The green dotted arrow indicates the region of the alignment from which the insertions were spliced out in each genome. The red dotted boxes indicate the regions of the virus genomes in which the 10.7 kb (**a**) and 5.5 kb (**b**) sequences of horsepox virus (thick black lines) are present or absent. The stars indicate the ITR junction sites in virus genomes

Interestingly, the 10.7-kb and the 5.5-kb deletions found, respectively, in the left and right ends of the genomes of all VACV strains [5] as well as in the Mulford 1902 strain [4] are also found partially or completely in the VK01 and VK8 genomes in the left and right ends, respectively. However, those deletions are not found in VK5, VK12 (only the right deletion is found), and VK2 (only the left deletion is found). In fact, the VK05 genome has the same genome structure (Fig. 2) and the highest identity to HSPV-MNR-76 across the whole genome, representing a true HSPV strain (Table 1). So far, MNR-76, isolated from Mongolian horses in 1976, and MNR, a synthetic recombinant horsepox virus, are the only extant strains of HSPV [5, 13].

VK08 genome is very similar to VK01, except for the absence of the 14.2-kb insertion (Fig. 2a, insert). VK02 genome has a 15-kb deletion near the very left end of the genome (Fig. 2a), resulting in the shortest ITRs in the VACV lineage (Table 1).

In conclusion, the re-assembly of the five VK genomes exposes the complex genetic diversity of the old smallpox vaccine genomes. We present evidence of the contemporaneous use of HSPV and HSPV-related viruses as the smallpox vaccine in the nineteenth century. The results also reveal that HSPV-related vaccines had been used in the USA at least 36 years before the Mulford 1902 strain. In the nineteenth century, vaccine seeds were constantly imported from Europe for smallpox vaccine production in the USA. Therefore, it is likely that HSPV and HSPV-related viruses were repeatedly introduced in the USA at that time and that similar vaccines were also manufactured and used in Europe in the nineteenth century [14].

## Data Availability

The datasets generated and/or analyzed during the current study are available in the Genbank accession numbers VK01 (BK013339), VK02 (BK013340), VK05 (BK013341), VK08 (BK013342), VK12 (BK013343), camelpox virus strain CMS (AY009089), variola virus strain Bgd75 (DQ437581), cowpox virus strains GRI90 (X94355), BriRed (AF482758), Aust1999 (HQ407377.1), HumBer07/1 (KC813509.1), GER90 (HQ420896), monkeypox virus strain DRC 07-0662 (JX878429), vaccinia virus strain Mulford 1902 (MF477237), horsepox virus strain MNR76 (DQ792504); vaccinia virus strains: WR (AY243312), Copenhagen (M35027), LC16m8 (AY678275), Serro 2 (KF179385), Cantagalo isolate CM-01 (KT013210), IHDW (KJ125439), Lister 107 (DQ121394), Dryvax clones DPP10 (JN654977), DPP11 (JN654978), DPP15 (JN654981), DPP20 (JN654985), DPP13 (JN654980), DPP21 (JN654986), DPP25 (KJ125438), ACAM2000 (AY313847), Tiantan clones TT8 (JX489135), TT5 (KC207811), Ectromelia virus strain Moscow (AF012825.2), Tashkent clones TKT3 (KM044309), and TKT4 (KM044310). Complete sequencing data are available through the SRA accession PRJNA561155.
